# Limited Evolution of Inferred HIV-1 Tropism while Viremia Is Undetectable during Standard HAART Therapy

**DOI:** 10.1371/journal.pone.0099000

**Published:** 2014-06-06

**Authors:** Guinevere Q. Lee, Winnie Dong, Theresa Mo, David J. H. F. Knapp, Chanson J. Brumme, Conan K. Woods, Steve Kanters, Benita Yip, P. Richard Harrigan

**Affiliations:** 1 BC Centre for Excellence in HIV/AIDS, Vancouver, BC, Canada; 2 University of British Columbia, Vancouver, BC, Canada; Centro Nacional de Microbiología - Instituto de Salud Carlos III, Spain

## Abstract

**Background:**

HIV patients on suppressive antiretroviral therapy have undetectable viremia making it impossible to screen plasma HIV tropism if regimen change is required during suppression. We investigated the prevalence and predictors of tropism switch from CCR5-using (“R5”) to non-CCR5-using (“non-R5”) before and after viral suppression in the initially therapy-naïve HOMER cohort from British Columbia, Canada.

**Methods:**

We compared pre-therapy and post-suppression viral genotypic tropism in patients who initiated on PI/NNRTI-based antiretroviral regimens between 1996-1999 (n = 462). Virologic suppression was defined as having two consecutive viral loads of <500 copies/mL, which was the sensitivity limit of most viral load assays at the time. Viral tropism was inferred by V3-loop-population-sequencing and geno2pheno_[coreceptor]_ with cutoff at 5.75% false positive rate (FPR).

**Results:**

When virologic suppression was defined as two-consecutive viral loads <500 copies/mL, 34 (9%) of the 397 patients with pre-therapy R5-virus switched to non-R5 at viral load rebound after a median of 19 months (IQR 8–41 months) of undetectable viremia. Duration of viral load suppression was not a predictor of switch, but lower CD4 count during suppression (median 400 versus 250 cells/mL) and an increased prevalence of pre-therapy non-R5 HIV by “deep” sequencing (median 0.2% versus 3.2%) were independently associated with switch (p = 0.03 and p<0.0001, respectively).

**Conclusion:**

R5-to-non-R5 tropism switches in plasma virus after undetectable viremia were relatively rare events especially among patients with higher CD4 counts during virologic suppression. Our study supports the use of pre-suppression tropism results if maraviroc is being considered during virologic suppression in this subgroup of patients.

## Introduction

HIV requires host cell coreceptors such as CCR5 and/or CXCR4 in addition to CD4 for cell-entry [1]. Viruses that use CCR5-molecules for cellular entry are referred to as “R5.” Viruses that use receptors other than CCR5, including the CXCR4-using “X4” viruses and the “dual/mixed-tropic” populations can collectively be termed “non-R5.” As CCR5-antagonists are only effective against R5 virus, viral tropism must be determined before prescribing this drug class. At the time of publication, maraviroc remains the first and only CCR5-anatognist approved for clinical use.

There are two approaches to determine plasma viral tropism commonly used in North America, phenotypic and genotypic. The phenotypic method offered by Monogram Biosciences in the United States, the Enhanced Sensitivity Trofile Assay (ESTA) [2], [3] utilizes *env* gene cloning and an infection-based assay [4], [5]. Genotypic methods are based on the amplification and population-sequencing of the V3-loop from patient viruses; “deep” sequencing technologies such as 454 (Roche) offers sensitivity comparable to phenotypic assays and outperforms population-sequencing in the detection of viral quasispecies for HIV tropism prediction and have recently gained popularity [6]. The V3-loop sequences are interpreted using prediction algorithms such as geno2pheno_[coreceptor]_ (g2p) [7]. However, both phenotypic and genotypic tropism prediction methods are limited to testing samples with sufficient plasma viral load typically above 250 HIV RNA copies/mL.

The majority of patients initiating highly active antiretroviral therapy (HAART) successfully suppress plasma viral load to undetectable levels (<50 copies/mL), making it impossible to perform viral tropism testing during viral suppression due to the detection limits of these plasma-based assays. This poses a challenge when considering CCR5-antagonist-based regimens as suitable options for treatment simplification or tolerability issues [8], [9]. To tackle this problem and to study the effect of HAART on viral tropism, investigators have focused on two main approaches: First, to examine tropism of integrated HIV proviral DNA in peripheral blood mononuclear cells (PBMC) during virological suppression, and second, to examine post-suppression plasma RNA tropism.

Studies on the effect of HAART on the evolution of viral tropism have focused primarily on comparing tropism of pre-therapy plasma viral RNA with tropism of viral DNA collected during suppression and observed concordance between 52–93% [10]–[16]. Studies on viremic patients have shown 71–100% tropism concordance between paired DNA and RNA samples [12], [15], [17]–[23]. Based on this limited evidence, DNA tropism testing of aviremic patients switching to maraviroc is currently recommended in several treatment guidelines [24]–[26] and is available both as a phenotypic and genotypic tests [22], [27].

However, the clinical utility of DNA tropism testing to predict maraviroc treatment outcomes in patients with low level viremia and/or undetectable viremia remains to be proven in randomized trials. Results from the smaller-scaled maraviroc “switch” studies demonstrated safety and efficacy [28]–[30], and it is hopeful that larger-scaled multicenter clinical trials such as the recruiting MARCH study [31] will shed more light on this knowledge gap. A second approach, the examination of pre-suppression HIV tropism from RNA, is considered in a few treatment guidelines [24], [26] based on small-scale studies that have shown limited evolution of plasma RNA tropism during HAART [10], [12], [32], [33].

The objective of this study was to compare plasma viral tropism between pre-therapy baseline and post-suppression time points in the absence of CCR5-anatagonist selective pressure. Our results provide relevant evidence to plasma-based tropism testing of pre-suppression samples for patients with undetectable viremia who wish to consider a CCR5 antagonist.

## Methods

### Ethics statement

This study was approved by the Providence Health Care Research Ethics Board; all participants provided written informed consent.

### Cohort and patient inclusion criteria

**Figure 1:**
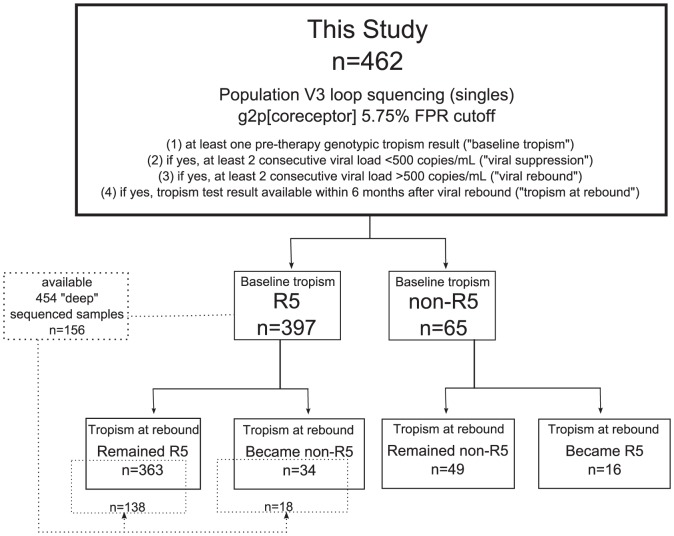
Study flow chart of our primary analysis: Virologic suppression definition: <500 copies/mL; geno2pheno_[coreceptor]_ FPR cutoff 5.75%.

HOMER is a well-characterized cohort consisting of 1188 treatment-naïve HIV-infected adults in British Columbia who initiated highly active antiretroviral therapy (HAART) between 1996 and 1999 [34]–[36]. As shown in Figure 1, a retrospective search of this database showed a subgroup of 462 individuals satisfied all four inclusion criteria of our primary analysis: individuals who (1) had at least one population-based sequencing tropism test result within six-months before their first exposure to HAART (“baseline tropism”); (2) had at least two consecutive samples collected with viral loads below 500 copies/mL post-HAART initiation (“viral suppression”); (3) after viral suppression had at least two consecutive samples collected with viral loads above 500 copies/mL (“viral rebound”); and (4) had genotypic tropism test results available from within six months after the date of viral rebound (“tropism at viral rebound”). Viral suppression was redefined as <50 copies/mL in part of our secondary analysis (n = 276).

### Laboratory methods

Baseline V3 sequences were determined as previously described [34]. For follow-up samples, HIV RNA was extracted from 0.5 mL plasma samples using the NucliSENS easyMag (bioMérieux). For population sequencing, a single-round (non-triplicate) reverse transcription and “nested” PCR were performed and sequencing reaction was performed with ABI 3730 DNA Sequencer and BigDye Terminator v3.1 Cycle Sequencing Kit (Applied Biosystems) as previously described [28], [37], [38]. Resulting chromatograms were base-called with in-house software RECall and aligned to a modified HXB2 V3-loop reference [39]. All Sanger sequences were deposited into GenBank (accession numbers EF637088-EF638007).

For “deep” sequencing, samples were put through triplicate reverse transcription reactions and “nested” PCR incorporating multiplex tags (MIDs A to L) as previously described [40], [41]. “Deep” sequencing reactions were performed with Genome Sequencer FLX System Standard kit with an average read length of 250 bases according to the manufacturer's supplied protocol (Roche/454 Life Sciences). A median of 1998 sequences (IQR 1575-2571) were obtained per sample. All population and 454 sequence data are available from the authors upon request, subject to review by the Providence Health Care Research Ethics Board.

### Data analysis

V3 sequences were interpreted by g2p_[coreceptor]_ [7] and tropism was inferred using cutoffs optimized to predict virologic response in the Phase III clinical trials of maraviroc [28], [37], [38], [40]–[42]. In our primary analysis, a sample was classified as “R5” by population sequencing if the false positive rate (FPR) predicted by g2p_[coreceptor]_ was >5.75%, and “non-R5” if FPR was ≤5.75%; a sequence was defined “R5” by “deep” sequencing if FPR was >3.5% and “non-R5” if ≤3.5% and a sample was considered non-R5 overall if ≥2% of sequences were found to be non-R5. In our secondary analysis, population-sequencing FPR cutoffs of 5%, 10%, 15% and 20% were explored. Statistical association analyses of demographic and clinical parameters were performed using SAS and/or GraphPad Prism 5. Pre and post-suppression nucleotide identity comparison in which base-mixtures were counted as concordant observations were performed with Python 3.3 scripting language.

## Results

### Prevalence and predictors of plasma HIV R5-to-non-R5 tropism switches

We first compared pre-therapy tropisms to post-suppression tropism at viral rebound. In our primary analysis, we defined virologic suppression as <500 copies/mL in order to suit the most common viral load assays' detection limit during the HOMER cohort enrollment period from 1996 to 1999. A total of 462 patients met the study criteria. Of the 397 subjects with pre-therapy R5 viruses, 34 were non-R5 after viral rebound (8.6%); of the 65 subjects with baseline non-R5 viruses, 16 were R5 after viral rebound (24.6%) (Figure 1). Subjects in this study experienced periods of suppressive HAART lasting a median of 19 (IQR 8–41) months. Pre- and post- therapy patient characteristics were summarized in Table 1.

**Table 1:** Baseline and post-therapy characteristics of all subjects (column 1 “All Subjects n = 462”) followed by the same dataset stratified by tropism switch categories determined by population-sequencing (columns 2–5).

	All Subjects	Remained non-R5	non-R5-to-R5	Remained R5	R5-to-non-R5	p-values^f^
	n = 462	n = 49	n = 16	n = 363	n = 34	
**Baseline characteristics**						
CD4 Median (IQR)	300 (150–430)	260 (140–430)	205 (65–295)	**310 (170**–**440)**	**255 (110**–**400)**	**0.099**
Log viral load Median (IQR)	5 (4.7–5)	5 (4.5–5)	5 (4.6–5)	**5 (4.7**–**5)**	**5 (4.5**–**5)**	**0.482**
Age (IQR)^a^	36 (31–43)	39 (33–45)	35 (31–37)	**36 (31**–**42)**	**38 (34**–**46)**	**0.079**
Gender, male (%)	390 (84%)	39 (80%)	15 (94%)	**306 (84%)**	**30 (88%)**	**0.803**
History of Injection Drug Use (%)	219 (47%)	18 (37%)	6 (38%)	**179 (49%)**	**16 (47%)**	**0.859**
**Post-therapy characteristics**						
Time to suppression in months (IQR)^b^	4 (2–14)	4 (2–6)	2 (1–13)	**4 (2**–**15)**	**5 (3**–**27)**	**0.289**
Duration of suppression in months (IQR)	19 (8–41)	20 (8–57)	15 (7–45)	**19 (8**–**41)**	**19 (6**–**33)**	**0.332**
CD4 at suppression (IQR)	390 (230–550)	380 (180–570)	235 (140–340)	**400 (240**–**570)**	**250 (160**–**530)**	**0.031**
CD4 at rebound (IQR)	390 (230–540)	340 (200–500)	265 (215–380)	**400 (255**–**550)**	**360 (180**–**510)**	**0.355**
Log viral load at rebound (IQR)	4.6 (3.7–5)	4.5 (4.1–5)	5.0 (4.6–5)	**4.6 (3.7**–**5)**	**4.7 (3.8**–**5)**	**0.710**
Adherence ≥95% (%)^c^	224 (49%)	23 (47%)	9 (56%)	**175 (48%)**	**17 (50%)**	**0.860**
PI -containing therapy (%)^d^	343 (74%)	39 (80%)	9 (56%)	**271 (75%)**	**24 (71%)**	**0.681** g
NNRTI-containing therapy (%)^e^	119 (26%)	10 (20%)	7 (44%)	**92 (25%)**	**10 (29%)**	**-**
AIDS-defining illness (%)	91 (20%)	12 (25%)	3 (19%)	**69 (19%)**	**7 (21%)**	**0.821**

a Age was categorized as follows: under 30, 30–39, 40–49, and 50 or more.

b Duration (in months) between HAART-initiation and virologic suppression defined as 500 copies/mL.

c Adherence ≥95% was defined as ≥95% compliance of prescription refills over first 12 months of therapy initiation.^d^ PI-containing therapy: drug category of a patient's first HAART therapy.

e NNRTI-containing therapy: drug category of a patient's first HAART therapy

f p-values were calculated based on comparisons between groups “Remained R5” and “R5-to-non-R5”.

g Fisher's Exact test comparing PI and NNRTI-containing therapy against Remained R5 and R5-to-non-R5 switch.

We then assessed associations with the clinical parameters listed in Table 1. None of the baseline characteristics was predictive of switch. Among post-therapy characteristics tested, only CD4 count at suppression (defined as the CD4 count test result obtained closest to the date of viral suppression) was found to be a predictor of switch. Subjects with R5-virus at baseline whose virus remained R5 after rebound (n = 363) had significantly higher CD4 counts at viral load suppression (median 400 cells/mL, IQR 240–570) than those who experienced a R5-to-non-R5 switch (median 250 cells/mL, IQR 160–530; p = 0.031, Mann-Whitney test). Importantly, duration of viral load suppression did not return as a predictor of switch. Non-R5-to-R5 switches and their associations with clinical parameters were not examined in this study because of unclear clinical importance.

**Figure 2:**
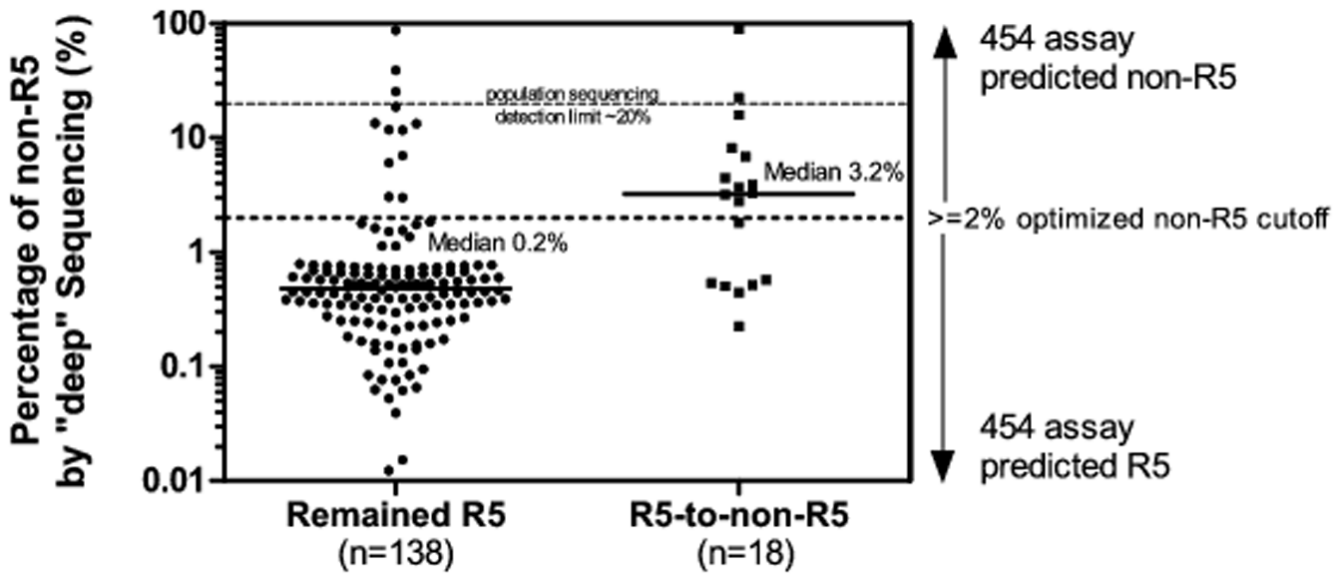
454 “deep” sequencing results of pre-therapy “R5” samples by population sequencing. In patients with pre-therapy baseline R5-viruses (n = 156), “deep” sequencing reveals that the prevalence of non-R5 viruses before starting HAART was a significant predictor of R5-to-non-R5 change (p<0.0001, Mann-Whitney test). Median non-R5 prevalence by “deep” sequencing among subjects who were tested as having R5 virus that remained R5 by population sequencing was 0.2% in comparison to 3.2% among those who had switched from R5 to non-R5. Horizontal bars indicate median values. For graphing and visualization purposes, values less than or equal to 1 were given randomized numbers between 0.01 and 0.8 such that samples with <1% non-R5 prevalence would randomly disperse across the plot from −2 to −0.1 log copies/mL. The dotted line at 2% non-R5 represents our group's optimized cutoff value (>2% non-R5 sequences) used for dichotomizing samples into non-R5 or R5. The dashed line at 20% represents the approximate sensitivity limit of population sequencing; five samples in this figure had %non-R5 above this sensitivity limit indicating 454 and population sequencing discordance. Detailed examination of these five samples suggested the high %non-R5 observed was a summation effect from multiple less prevalent non-R5 sequences in four, and was due to random sampling bias in one sample (Table S1).

Pre-therapy baseline “deep” sequencing results were available for a subset of patients (n = 156) with baseline R5 virus by population sequencing (Figure 1). In these patients a median of 0.2% (IQR 0.1–0.7%) of detected sequences were inferred to be non-R5. Using this method, 11/18 (61%) of individuals who switched tropism from R5 at baseline to non-R5 after viral rebound by population sequencing were called “non-R5” at baseline by “deep” sequencing (≥2% “non-R5” sequences), compared to 12/138 (9%) of individuals who did not switch tropism (p<0.0001, Chi-square test). Also, an increased prevalence of non-R5 viruses in pre-therapy samples was significantly associated with R5-to-non-R5 tropism switches (p<0.0001, Mann-Whitney test, Figure 2). This suggests that dichotomized results from the “deep” sequencing tropism prediction assay of pre-therapy samples also predicted tropism switches after viral rebound.

### R5-to-non-R5 tropism switches during period of detectable viremia

In the analysis described above, pre-HAART viral tropism was compared to post-suppression viral tropism. However, periods of detectable viremia following the start of HAART but before viral suppression (R5-to-non-R5 n = 34 median 5 months IQR 3-27, Table 1) and periods of detectable viremia post-suppression before the first available tropism results (R5-to-non-R5 n = 34, median 4 months IQR 3–6) might have provided sufficient time for viral evolution and a chance for non-R5 HIV populations to be selected, which would lead to an over-estimation of our observed switch prevalence over the period of suppressive-HAART. To address this study limitation, we looked for and genotyped any archived plasma samples or tropism test results collected immediately before and/or after viral suppression for the 34 subjects who experienced R5-to-non-R5 switches.

Appropriate pre-suppression samples or tropism test results were available for 21/34 (62%) of the subjects. Of these, non-R5-viruses were detected in 11 before viral load suppression occurred; “deep” sequencing results available for 8 of the 11 subjects revealed median prevalence of 3% non-R5 (IQR 1-9%) at pre-therapy baseline. Appropriate untested post-suppression plasma sampled before our definition of “first tropism test result available after viral rebound” were available for 3/34 (9%) of the subjects. Of these, one subject harbored post-suppression R5-virus; “deep” sequencing showed 1% non-R5 at pre-therapy baseline. In summary, 12/34 (35%) of the initially observed R5-to-non-R5 tropism switches could be explained by switches that occurred during periods of detectable viremia.

### Secondary analysis: Exploration of other cutoff values and nucleotide sequence comparison

As a sensitivity analysis reflecting a more modern definition of virologic suppression, the analysis was repeated with suppression defined as <50 copies/mL (FPR cutoff 5.75%, n = 276). Results were similar to our primary analysis: R5-to-non-R5 switch occurred in 13/247 (5%) and non-R5-to-R5 switch occurred in 6/29 (21%) patients.

Sensitivity analysis of different g2p_[coreceptor]_ FPR cutoffs 5%, 10%, 15% and 20% in combination with viral suppression defined as 500 or 50 copies/mL showed an underestimation of R5-to-non-R5 switches at lower FPR cutoffs: When suppression was defined as <500 copies/mL, R5-to-non-R5 switches occurred at 7%, 10%, 14%, 17% prevalence, and non-R5 switches occurred at 27%, 24%, 25%, 22% respectively. When suppression was defined as <50 copies/mL, R5-to-non-R5 switches occurred at 5%, 8%, 12%, 15%, and non-R5 switches occurred at 24%, 24%, 29%, 22% respectively.

Next, we compared pre-therapy and post-suppression V3-loop population-sequences. Phylogenetic comparison by neighbor-joining tree shows that most sequence-pairs clustered together but this method was limited by the short sequence length (Figure S1); per-position nucleotide identity comparison shows a low number of base discordance between sequences obtained from the same individuals from the two time points (median nucleotide discordance count was 0, IQR 0–2, min 0 max 19; average V3 loop length 105 nucleotides).

## Discussion

In this study we compared the pre-HAART HIV RNA viral tropism with the viral tropism after viral rebound in the plasma of individuals of the British Columbia HOMER cohort. In our primary analysis, we reported R5-to-non-R5 tropism switches in less than 9% of subjects over a median of 19 months of pVL suppression on HAART. This switch was predicted by a higher percentage prevalence of non-R5 species at pre-therapy baseline and a lower CD4 count during viral suppression, but not by the duration of viral load suppression.

Previous smaller-scale studies (ranging from n = 18 to 36 pre-therapy R5 cases) reported pre-therapy -R5 to post-therapy-non-R5 tropism change in 5–25% of their subjects [10], [12], [32], [33], [43], compared to 20% (n = 30) in untreated patients [10]. Our study population was at least ten times larger than any previous studies and our observation fell within the range of previous observations. As such, this study has provided additional supporting evidence for clinical management guidelines [24], [26] on the use of pre-suppression tropism results to infer eligibility of initiating a maraviroc-containing regimen during suppression.

Furthermore, our results suggest that the relative prevalence of non-R5 viruses at baseline detected by “deep” sequencing could partially explain eventual tropism switches observed in population sequencing results. In 61% of cases, patients whose HIV tropism switched from R5 to non-R5 would have already been classified as non-R5 at baseline by the more sensitive deep sequencing test.

However, the explanation for the observed association with low CD4 counts during suppression is less clear. It is interesting to note that several studies have reported 2–6 times lower nadir and/or baseline CD4 count as the only association identified with tropism switches [10], [16], whereas another study observed a two-fold lower nadir CD4 count in patients hosting DNA-tropism-based non-R5 viruses compared to those hosting R5 viruses [44] while other studies were unable to find CD4 count associations of this kind [12], [45]. Selection pressures that lead to a R5-to-non-R5 tropism switch in the absence of CCR5-antagonists remain poorly understood.

There were a number of limitations to this study. The first is our study's definition of “undetectable viral load” and “viral suppression” of <500 copies/mL. Previous studies showed that prolonged periods of low level viremia (LLV, roughly defined as 50–500 copies/mL) allowed for viral evolution defined as increasing numbers of drug resistance mutations and/or HLA-escape mutations [45]–[53]. Our current definition could lead to an over-estimation of the prevalence of tropism switch if results were to apply to the current definition of undetectable viremia which is typically 20–50 copies/mL [54]. Indeed, our secondary analysis showed that when suppression was redefined to <50 copies/mL, we detected a lower prevalence (from 9% to 5%) of R5-to-non-R5 switches.

A second study limitation was our choice of pre-HAART tropism as the comparator. Although the length of time between HAART initiation and viral suppression was not significantly associated with tropism switch, some patients in this study achieved viral suppression over one year after therapy initiation, allowing active viral replication and potential viral evolution. Indeed, when we tested additional samples collected immediately before or after viral load suppression from these individuals, we observed 35% of the patients who experienced R5-to-non-R5 switches could be explained by switches during the initial decline in viremia prior to suppression or by post-suppression switches.

A third study limitation was genotypic tropism determination methods' limited sensitivity/specificity relative to the “true” viral tropism or to the clinical outcomes of individuals receiving CCR5-antagonist-based regimens. It is important to understand that even ESTA, a phenotypic tropism determination assay, is limited by sensitivity and specificity [55], [56]. While a 100% sensitive method to determine viral tropism does not exist because there is no distinct gold standard for HIV viral tropism [26], [57], population-sequencing-based genotypic tropism prediction has been reported to predict maraviroc-based regimen virological outcome [28], [37] and have a sensitivity of 67.4% and specificity of 92.6% against a phenotypic assay [37], which implies that our reported prevalence of post-HAART tropism change can only be taken as an estimation.

Overall, this study showed that R5-to-non-R5 tropism switches after periods of suppressive-HAART were relatively rare events, especially in patients with higher CD4 counts during suppression and/or patients with a lower prevalence of circulating non-R5 quasispecies in their baseline plasma samples. Since a large proportion of our observed cases of tropism switches occurred during periods of detectable viremia, the last tropism test before suppression could be more ideal than a pre-HAART tropism test in predicting tropism switch after viral rebound. Furthermore, our “deep” sequencing results reinforce the increased sensitivity of “deep” sequencing assay as a prediction tool for viral tropism. These results also suggest that pre-HAART plasma RNA “deep” sequencing tropism results, reported either as the percentage non-R5 prevalence or dichotomized as R5/non-R5, could serve as yet another complementary test in addition to DNA tropism predictions for patients with undetectable viremia. Future studies should examine if pre-HAART or pre-suppression RNA R5 tropism is a predictor of clinical outcome in patients who switched into maraviroc-containing regimens during viral suppression.

## Supporting Information

Figure S1 **Phylogenetic analysis.** Neighbor-joining tree of paired V3-loop sequences from pre-therapy (baseline) and post-suppression (rebound) time points. Individual sequences were labeled in this format: patient-identifier_timepoint.
(PDF)

Table S1 **Five samples had “>20%non-R5” by 454 despite being predicted “R5” by population sequencing (** **Figure 2** **, main text).** This table offers explanations for the discordance between 454 and population sequencing.
(DOCX)
