# Supplementary material for: Limited Evolution of Inferred HIV-1 Tropism while Viremia Is Undetectable during Standard HAART Therapy
Source: PLoS One. 2014 Jun 6;9(6):e99000. doi: 10.1371/journal.pone.0099000 (PMC4048224; doi:10.1371/journal.pone.0099000)
Supplement: Table S1 — Five samples had “>20%non-R5” by 454 despite being predicted “R5” by population sequencing ( Figure 2 , main text). This table offers explanations for the discordance between 454 and population sequencing. (DOCX) [file pone.0099000.s002.docx]

**Supplementary Table 1.** Five samples had “>20%non-R5” by 454 despite being predicted “R5” by population sequencing (Figure 2, main text). In samples A, B, C, and D, the most-prevalent 454 observations matched perfectly with the observed population sequence; however, multiple variants that ranked 2nd to 5th in prevalence had g2p fpr below 3.5%. Therefore, the high %non-R5 we observed was a summation effect from multiple less prevalent non-R5 sequences that was “missed” by population sequencing. In sample E, population sequence did not match the most prevalent 454 observation, but instead matched with the 4th and 5th most prevalent 454 observations. The low fpr observed in the top-three 454 observations was mainly a contribution of the “R” at position 25 that was detected by 454 but was by chance “missed” by population sequencing method. Therefore, for this sample, “random sampling bias” would be the most logical explanation to the discrepancy observed between 454 and population method.

| **Sample** | **Method** | **% Total** | **%nonR5** | **g2p fpr%** | **Translated amino acid sequences** |
| --- | --- | --- | --- | --- | --- |
| A | population | - |  | 56.1 | CTRPNNNTRKGIHLGPGQALYTT-EVIGDIRQAHC  I G Y ^a^see footnote |
|  | 454-Top(1st) | 25.1% | 39.1% | 66.2 | CTRPNNNTRKGIHLGPGQALYTT-EVIGDIRQAHC |
|  | 454-2nd | 21.4% |  | 1.8 | CTRPNNNTRKGIYLGPGRRLYTT-RVIGDIRQAHC |
|  | 454-3rd | 7.1% |  | 1.7 | CTRPNNNTRKGIYLGPGRALYTT-RVIGDIRQAHC |
|  | 454-4th | 3.6% |  | 59.8 | CTRPNNNTRKGIHLGPGQALYTT-EVIGDIRQAYC |
|  | 454-5th | 2.9% |  | 1.7 | CTRPNNNTRKGIYLGPGRALYTT-RVIGDIRKAHC |
| B | population | - |  | 61.4 | CTRPNNNTRKGIHMGPGGAFFATGEIIGNIRQAHC  A |
|  | 454-Top(1st) | 34.8% | 25.3% | 76.5 | CTRPNNNTRKGIHMGPGGAFFATGEIIGNIRQAHC |
|  | 454-2nd | 16.8% |  | 2.5 | CTRPNNNTNKSIHMGARRAFHATEKIIGNVKQAHC |
|  | 454-3rd | 3.7% |  | 61.4 | CTRPNNNTRKGIHMGPGGAFFATGGIIGDIRQAHC |
|  | 454-4th | 2.7% |  | 3.8 | CTRPNNNTSKSIHMGARRAFHATEKIIGNVKQAHC |
|  | 454-5th | 1.9% |  | 3.8 | CTRPNNNTSKSIHMGARRAFHATEKIIGNVKQAHC |
| C | population | - |  | 7.1 | CTRPSNNTRKGIHIGPGRAVYATEQITGDTRQAHC  R FF GE I |
|  | 454-Top(1st) | 29.4% | 22.5% | 9.6 | CTRPSNNTRKGIHIGPGRAFYATEQITGDIRQAHC |
|  | 454-2nd | 21.3% |  | 20.9 | CTRPSNNTRKGIHIGPGRAFYATGEITGDTRKAHC |
|  | 454-3rd | 9.1% |  | 1.1 | CTRPNNNTRKGIRIGPGRAVIATEKITGDIRKAHC |
|  | 454-4th | 4.0% |  | Error^b^ | CTRPSNNTRKGIHIGPGRAFYATEK*QEI------ |
|  | 454-5th | 3.5% |  | 1.3 | CTRPNNNTRKGIRIGPGRAVIATEKITGDIRQAHC |
| D | population | - |  | 7.8 | CTRPGNNTRRSIGIGPGRAFFTTGDIIGDIRKAHC  V Q |
|  | 454-Top(1st) | 54.6% | 89.2% | 3.4 | CTRPGNNTRRSIGIGPGRAFFTTGDIIGDIRKAHC |
|  | 454-2nd | 12.2% |  | 3.4 | CTRPGNNTRRSIGIGPGRAFFTTGDIIGDIRKAHC |
|  | 454-3rd | 7.4% |  | 3.4 | CTRPGNNTRRSIGIGPGRAFFTTGDIIGDIRKAHC |
|  | 454-4th | 1.9% |  | 0.5 | CTRPNNNTRRGIHIGLGRAVYVRRKIIGDIRQAHC |
|  | 454-5th | 1.1% |  | 2.2 | CTRPGNNTRRSIGVGPGRAFFTTGDIIGDIRKAHC |
| E | population | - |  | 17 | CTRPNNNTRKSIHMGPGKACYTTGEIIGDIRQAHC  G R F G |
|  | 454-Top(1st) | 60.0% | 87.1% | 1.7 | CTRPNNNTRKGIHIGPGRTWYTTRRIIGDIRKAHC |
|  | 454-2nd | 5.3% |  | 1.7 | CTRPNNNTRKGIHIGPGRTWYTTRRVIGDIRKAHC |
|  | 454-3rd | 4.3% |  | 1.7 | CTRPNNNTRKGIHIGPGRTWYTTRRIVGDIRKAHC |
|  | 454-4th | 2.2% |  | 26.9 | CTRPNNNTRKSIHMGPGKAFYTTGEIIGDIRQAHC |
|  | 454-5th | 2.2% |  | 21.2 | CTRPNNNTRKGIHMGPGKAFYTTGEIIGDIRQAHC |

^a^ Mixtures detected in population sequencing that could be translated into >1 amino acids were translated accordingly, and were represented in this table vertically in the population sequence rows. For example, in sample A, at the second-to-last amino acid position, population sequencing indicated a nucleotide mixture that could be translated into both amino acids H and Y.

^b^ Error in g2p algorithm due to missing sequence information in the 3’ end and an ambiguous base N reported by 454.
