# Supplementary material for: Limited Evolution of Inferred HIV-1 Tropism while Viremia Is Undetectable during Standard HAART Therapy
Source: PLoS One. 2014 Jun 6;9(6):e99000. doi: 10.1371/journal.pone.0099000 (PMC4048224; doi:10.1371/journal.pone.0099000)

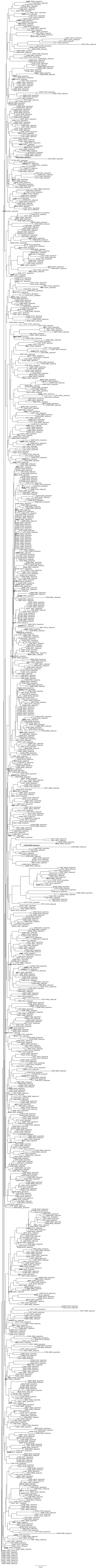

Supplement: Figure S1 — Phylogenetic analysis. Neighbor-joining tree of paired V3-loop sequences from pre-therapy (baseline) and post-suppression (rebound) time points. Individual sequences were labeled in this format: patient-identifier_timepoint. (PDF) [file pone.0099000.s001.pdf]
